# Comparison of infection and complication rates associated with transvenous vs. subcutaneous defibrillators in patients with stage 4 chronic kidney disease: a multicenter long-term retrospective follow-up

**DOI:** 10.3389/fcvm.2024.1397138

**Published:** 2024-04-10

**Authors:** Fabian Schiedat, Benjamin Meuterodt, Magnus Prull, Assem Aweimer, Michael Gotzmann, Stephen O’Connor, Christian Perings, Johannes Korth, Thomas Lawo, Ibrahim El-Battrawy, Christoph Hanefeld, Andreas Mügge, Axel Kloppe

**Affiliations:** ^1^ Department of Cardiology and Angiology, University Hospital Bergmannsheil Bochum of the Ruhr-University Bochum, Bochum, Germany; ^2^ Department of Cardiology and Angiology at Marienhospital Gelsenkirchen, Academic Hospital of the Ruhr University Bochum, Gelsenkirchen, Germany; ^3^ Department of Cardiology, Electrophysiology, Pneumology and Intensive Care Medicine, St. Marien-Hospital Luenen, Academic Hospital of the University Muenster, Luenen, Germany; ^4^ Department of Cardiology, Augusta Hospital Bochum, Academic Hospital of the University Duisburg-Essen, Bochum, Germany; ^5^ Department of Cardiology, Katholische Kliniken Bochum of the Ruhr University Bochum, Bochum, Germany; ^6^ Department of Biomedical Engineering, City, University of London, London, United Kingdom; ^7^ Department of Nephrology, University Hospital Essen, University of Duisburg-Essen, Essen, Germany; ^8^ Department of Cardiology, Elisabeth Hospital Recklinghausen, Recklinghausen, Germany; ^9^ Department of Molecular and Experimental Cardiology, Institut für Forschung und Lehre (IFL), Ruhr-University Bochum, Bochum, Germany

**Keywords:** sudden cardiac death (SCD), implantable cardiac defibrillator (ICD), S-ICD, device infection, device complication, chronic kidney disease

## Abstract

**Background:**

Patients with progressive chronic kidney disease (CKD) are at higher risk of infections and complications from cardiac implantable electronic devices (CIED). In patients with a primary or secondary prophylactic indication, implantable cardiac defibrillators (ICD) can prevent sudden cardiac deaths (SCD). We retrospectively compared transvenous-ICD (TV-ICD) and intermuscularly implanted subcutaneous-ICD (S-ICD) associated infections and complication rates together with hospitalizations in recipients with stage 4 kidney disease.

**Methods:**

We retrospectively analyzed 70 patients from six German centers with stage 4 CKD who received either a prophylactic TV-ICD with a single right ventricular lead, 49 patients, or a S-ICD, 21 patients. Follow-Ups (FU) were performed bi-annually.

**Results:**

The TV-ICD patients were significantly older. This group had more patients with a history of atrial arrhythmias and more were prescribed anti-arrhythmic medication compared with the S-ICD group. There were no significant differences for other baseline characteristics. The median and interquartile range of FU durations were 55.2 (57.6–69.3) months. During FU, patients with a TV-ICD system experienced significantly more device associated infections (*n* = 8, 16.3% vs. *n* = 0; *p* < 0.05), device-associated complications (*n* = 13, 26.5% vs. *n* = 1, 4.8%; *p* < 0.05) and device associated hospitalizations (*n* = 10, 20.4% vs. *n* = 1, 4.8%; *p* < 0.05).

**Conclusion:**

In this long-term FU of patients with stage 4 CKD and an indication for a prophylactic ICD, the S-ICD was associated with significantly fewer device associated infections, complications and hospitalizations compared with TV-ICDs.

## Introduction

Implantable cardioverter defibrillators (ICD) are indicated for heart failure (NYHA II or III) with a reduced left-ventricular ejection fraction (LV-EF) ≤ 35% despite optimal medical treatment for more than 3 months, as well as patients who have recovered from a hemodynamic unstable ventricular arrhythmias without a reversible cause (1, 2). Patients are also required to have a life expectancy of more than one year with a good quality survival. Patients without the need for bradycardia pacing, anti-tachycardia pacing or cardiac resynchronization therapy (CRT) may receive either a subcutaneous implantable cardioverter defibrillator (S-ICD) or a transvenous implantable cardioverter defibrillator (TV-ICD) (1).

With progressive chronic kidney disease (CKD), patients are at an increased risk of cardiovascular mortality and morbidity, which still exists after correction for typical concomitant risk factors (3). A main trigger is believed to be an inflammatory reaction in CKD (4) which has been identified as an independent risk factor for cardiac implantable electronic device (CIED) infections and complications (5). Patients with a glomerular filtration rate (GFR) < 30 ml/min/1.73 m^2^ are a CKD subgroup exposed to a risk for infections and lead complications (6) whilst those with a GFR 15–29 ml/min/1.73 m^2^ for at least three months are defined as stage 4 CKD and at even higher risk (7).

The S-ICD has been associated with lower rates for lead related complications during long-term follow-up (FU) while demonstrating comparable safety and efficacy compared to TV-ICDs (8). S-ICD device-related infections are less frequent as there are no leads on or in the heart. If they do occur, S-ICD infections can be handled conservatively without the need for urgent removal as is the case with TV-ICDs, where transvenous lead extraction (TLE) is associated with high mortality and morbidity (9, 10). Very limited long-term data on infection and complication rates in patients with progressive CKD receiving an ICD have been published. There is however no comparative data in this compromised cohort of CKD patients between TV-ICD and S-ICD recipients. The aim of the present study was to compare long-term device associated safety and outcomes in patients with stage 4 CKD who received either an TV-ICD or S-ICD with a single right ventricular lead.

## Methods

Out of the population of patients undergoing TV-ICD or S-ICD implantation, those with stage 4 CKD, GFR 15–29 ml/min/1.73 m^2^ for at least 3 months, were included in this retrospective analysis from six experienced centers in Germany (University Hospital Bergmannsheil Bochum, University Hospital Katholische Kliniken St. Josef Bochum, Marienhospital Gelsenkirchen, Augusta Hospital Bochum, Marien-Hospital Luenen and Elisabeth Hospital Recklinghausen). Patients on hemodialysis were excluded from this study as hemodialysis is an independent risk factor for device infection and complication (11). Patients with stage 5 CKD without dialysis were excluded as well, as there is a very high likelihood for hemodialysis during FU compared to stage 4 CKD (12).

The devices were implanted between 2012 and 2020. We obtained informed consent from all patients. The study protocol conforms to the ethical guidelines of the 1964 Declaration of Helsinki and its later amendments. It was approved by the local ethics committees of the Ruhr-University, Bochum as the leading ethics committee (Register 22-7593-BR). All patients had indications for a cardiac defibrillator implantation following the guidelines and a life-expectancy >1 year (1, 2).

### Implantation procedure

Implantation was performed under local anesthesia combined with deep sedation. Intravenous antibiotic prophylaxis as a single dose was given prior to the procedure with 2 grams of cefazolin or alternatively 1 gram of vancomycin. The decision to implant either an TV-ICD or S-ICD was done according to operators' discretion.

For TV-ICD implantation, the pulse generator was placed in a left-sided, pre-pectoral, sub-fascial position. The lead was placed via the cephalic, axillary or subclavian vein, in a mid-septal right ventricular position and sutures were applied twice at the sleeve and once at the generator. An inter-muscular approach was used for the S-ICD implantation (13). The device (Emblem S-ICD, Model 209, Boston Scientific, Marlborough, MA, USA or SQ-RX 1010, Cameron Health, San Clemente, CA, USA) was placed in a left postero-lateral position between the anterior surface of serratus anterior and the posterior surface of the latissimus dorsi over the left fifth or sixth rib in an intermuscular space between the mid and posterior axillary lines. The S-ICD electrode was implanted left parasternally via a two- incision technique and sutures were applied at all incision sites. The device was placed in the pocket and sutures were applied at both sides of the generator. Induced arrhythmia conversion testing was performed once the electrode and pulse generator were in their respective positions prior to closure. Induction of ventricular fibrillation was facilitated by 50 Hz stimulation burst between the shock coil and pulse generator and terminated by a 65-Joule shock thereby giving a 15-Joule safety margin. In case of non-conversion of the first induction, the position of the electrode and pulse generator were checked with fluoroscopy and repositioned, if necessary. Testing was then repeated with the option for reversing shock polarity. Complications that were life-threatening, with permanent adverse sequelae or resulted in death were defined as major complication. All other procedure related complications were defined as minor complications.

### Patient follow-up

All patients were seen prior to discharge for wound control as well as device interrogation and programming. All three S-ICD sensing vectors were tested in a supine and standing positions. Patients were followed up bi-annually in the outpatient department. Device interrogation, signs of device associated complications and infection, as well as hospitalizations, were documented. Where a patient failed to attend their FU, they and/or their family physician were contacted to confirm whether they were alive. Complications, hospitalizations and other events were thereby ascertained and documented. If hemodialysis was required during FU, the FU period for these patients ended with the day of the first hemodialysis treatment.

### Data collection

Data has been collected continuously but analyzed retrospectively and audited with hospitals' clinical information system.

### Statistical analysis

All statistical analyses were performed using IBM SPSS Statistics Version 28.0.0 on Mac. Categorial variables were expressed as frequencies and percentages (normal distribution) or median and interquartile range (non-normal distribution). Continuous variables were stated as mean ± standard deviation. Assessment of descriptive statistics and baseline characteristics was done using linear regression. If necessary, Chi-quadrat test was used for normally distributed, non-normally distributed and binary data with linear trends. Time to primary endpoints were performed using Kaplan–Meier analyses and compared with the log-rank test.

## Results

### Patient population and implant procedure

A total of 70 patients from six German centers with stage 4 CKD and indication for ICD implantation were included in this study. All procedures were performed by six experienced operators, one at each center. During the same period of time, a total of 2,757 prophylactic TV-ICDs with single right ventricular lead and S-ICD implantations were performed at the six participating centers. Of the 70 patients with stage 4 CKD in this study, 49 patients received a TV-ICD with a single right ventricular, single-coil lead, and 21 patients received a S-ICD. The indication was primary prophylactic in 73.5% of the TV-ICD and 85.7% of the S-ICD population (*p* = 0.267). The TV-ICD population was significantly older (73.0 (68.5–78.0) years vs. 59.0 (50.0–67.5) years; *p* < 0.01) than the S-ICD population. In addition, the TV-ICD cohort had a significantly higher burden of atrial fibrillation and received more anti-arrhythmic medication and cardiac glycosides. There were no significant differences in other baseline characteristics. All baseline characteristics are shown in Table 1. All S-ICD implantation procedures were successful with a 15-Joule safety margin being established from induced arrhythmia conversion testing, with no patients requiring an electrode position change or retest. Implant duration was not significantly different between defibrillator types.

**Table 1:** Baseline and procedure characteristics.

	TV-ICD (49)	S-ICD (21)	*p*
Age	73.0 (68.5–78.0)	59.0 (50.0–67.5)	<0.01
BMI (kg/m^2^)	27.1 (24.2–30.4)	27.6 (24.6–33.9)	0.166
Female gender, *n* (%)	10 (20.4)	5 (23.8)	0.755
Primary prophylactic indication, *n* (%)	36 (73.5)	18 (85.7)	0.267
Anemia, *n* (%)	24 (49.0)	7 (33.3)	0.189
Hyperparathyroidism, *n* (%)	1 (2.0)	0	0.517
History of renal transplant, *n* (%)	0	0	-
Ischemic cardiomyopathy, *n* (%)	30 (61.2)	13 (61.9)	0.258
NYHA class	2.1 ± 0.9	1.4 ± 0.5	0.102
Coronary artery disease, *n* (%)	40 (81.6)	16 (76.2)	0.608
Myocardial infarction, *n* (%)	28 (57.1)	7 (33.3)	0.103
History of heart surgery, *n* (%)	13 (26.5)	5 (23.9)	0.815
Atrial arrhythmia, *n* (%)	25 (51.0)	2 (9.5)	<0.05
Arterial hypertension, *n* (%)	44 (89.8)	17 (81.0)	0.318
Diabetes, *n* (%)	28 (57.1)	10 (47.6)	0.235
Hyperlipoproteinemia, *n* (%)	30 (61.2)	13 (61.9)	0.958
Chronic obstructive lung disease, *n* (%)	14 (28.6)	2 (9.5)	0.084
Stroke of ischemic and non-ischemic etiology, *n* (%)	2 (4.1)	1 (4.8)	0.899
Liver disease, *n* (%)	0	1 (4.8)	0.128
Peripheral artery disease, *n* (%)	9 (18.4)	5 (23.8)	0.608
History of vascular surgery, *n* (%)	4 (8.2)	3 (14.3)	0.441
Potassium (mmol/L)	4.4 ± 0.7	4.6 ± 0.7	0.215
GFR (ml/min/1.73 m^2^)	24.1 ± 8.4	23.7 ± 7.0	0.862
Creatinine (mg/dl)	2.8 ± 0.5	2.9 ± 0.4	0.352
Urea (mg/dl)	62.1 ± 40.2	61.0 ± 35.3	0.991
Hemoglobin (g/dl)	12.5 ± 1.7	13.3 ± 2.4	0.132
CRP (mg/dl)	2.0 ± 2.4	1.4 ± 1.3	0.243
Betablocker, *n* (%)	43 (87.8)	19 (90.5)	0.747
ACE inhibitor/ARB, *n* (%)	40 (81.6)	16 (76.2)	0.608
MRA, *n* (%)	24 (49.0)	11 (52.4)	0.798
Diuretics, *n* (%)	47 (95.9)	18 (85.7)	0.133
Anti-arrhythmic medication, *n* (%)	25 (51.0)	2 (9.5)	<0.05
Cardiac glycosides, *n* (%)	13 (26.5)	0	<0.05
ASS, *n* (%)	32 (65.3)	14 (66.7)	0.914
DAPT, *n* (%)	18 (36.7)	5 (23.8)	0.298
OAK, *n* (%)	23 (47.0)	5 (23.8)	0.137
Corticosteroid, *n* (%)	4 (8.2)	1 (4.8)	0.619
Immunosuppression medication, *n* (%)	0	0	-
Insulin, *n* (%)	17 (34.7)	5 (23.8)	0.378
LV-EF (%)	29.0 (24.5–33.5)	28.7 ± 6.4	0.480
Implant duration (min)	55.0 (44.0–71.0)	77.3 ± 19.6	0.136
Minor perioperative complications, *n* (%)	6 (12.2)	1 (4.8)	<0.05
Minor perioperative complications requiring intervention, *n* (%)	1 (2.0)	0	0.689

TV-ICD, transvenous ICD; S-ICD, subcutaneous ICD; BMI, Body Mass Index; NYHA, New York Heart Association; GFR, glomerular filtration rate; CRP, C-reactive protein; ACE, angiotensin converter enzyme; ARB, angiotensin II receptor antagonists; MRA, mineralocorticoid receptor antagonist; ASS, acetylsalicylic acid; DAPT, dual anti-platelet therapy; OAK, oral anti-coagulant therapy; LV-EF, left ventricular ejection fraction.

There were no major complications during the intra-operative or the hospitalization period for implantation in either group. Minor perioperative complications occurred in 6 (12.2%) and 1 (4.7%) patients (*p* < 0.01) in the respective TV-ICD and S-ICD groups. There was no significant difference between groups for patients requiring an intervention for these complications (TV-ICD: *n* = 1, 2.0% vs. S-ICD: *n* = 0, *p* = 0.689). In the TV-ICD group, 1 (2.0%) patient experienced a minor vascular complications, 2 (4.1%) patients a pocket hematoma, 2 (4.1%) patients a pneumothorax and 1 (2.0%) patient a lead related complication due to undersensing within 2 weeks of implantation. Only the latter patient required an intervention, an RV lead revision. All other patients were managed conservatively without sequelae. In the S-ICD group, 1 (4.8%) patient had a pocket hematoma before discharge. This was handled conservatively without the need for a pocket revision.

All implant procedure characteristics and complications are shown in Table 1.

### Follow-up

**Figure 1:**
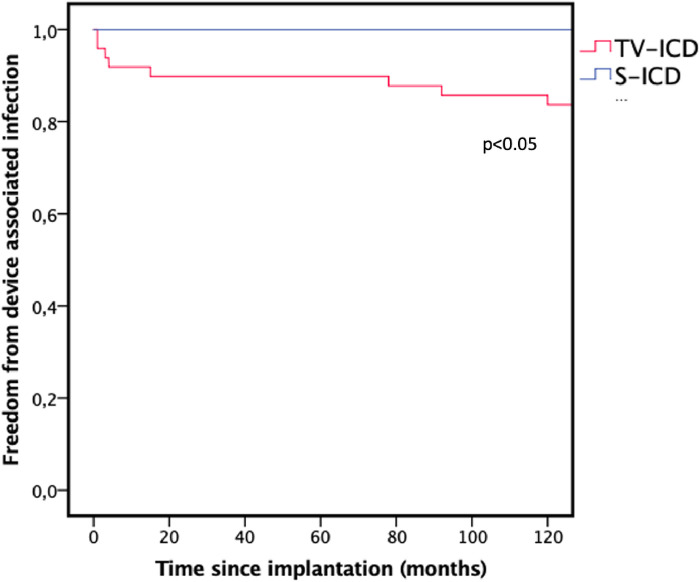
Kaplan–Meier graph illustrating the freedom from device associated infections during follow-up. TV-ICD, transvenous ICD; S-ICD, subcutaneous ICD.

Duration of FU was 55.1 (48.2–64.4) months in the TV-ICD group and 55.4 (46.8–75.0) months in the S-ICD group (*p* = 0.790). During FU, CIED associated infections occurred significantly more often in patients in the TV-ICD group (TV-ICD: *n* = 8, 16.3% vs. S-ICD: *n* = 0; *p* < 0.05). This is illustrated in Figure 1. Time to device infection in the TV-ICD group was 29.2 ± 10.6 months with all cases requiring TLE and generator removal.

**Figure 2:**
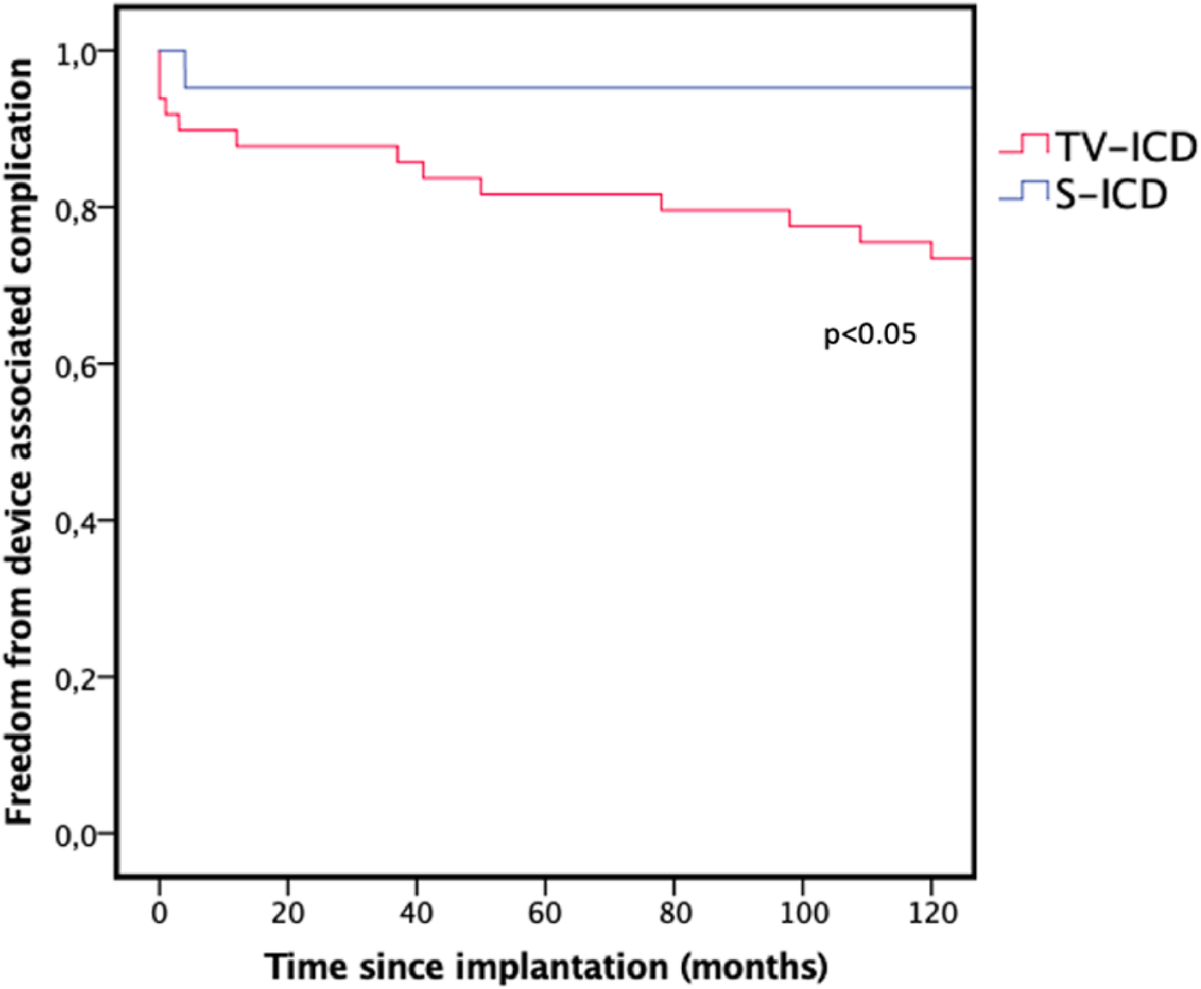
Kaplan–Meier graph illustrating the freedom from device associated complication during follow-up. TV-ICD, transvenous ICD; S-ICD, subcutaneous ICD.

CIED-associated complications without infection again occurred significantly more frequently in the TV-ICD group (TV-ICD: *n* = 13, 26.5% vs. S-ICD: *n* = 1, 4.8%, *p* < 0.05) as illustrated in Figure 2. The complications in the TV-ICD group were lead fracture with inadequate therapy delivery due to oversensing (*n* = 3), loss of right ventricular sensing <3 mV (*n* = 2), loss of capture (*n* = 2), drop in impedance below 250 Ohm (*n* = 1), increase in impedance above 1,500 Ohm (*n* = 2) and other non-infectious pocket or generator related complications (*n* = 3). Lead related complications in the TV-ICD group therefore occurred in 10 (20.4%) patients compared with 1 (4.8%) patient in the S-ICD group due to generator dislodgement during FU which required repositioning. The difference in lead related complications was significant (*p* < 0.05).

Overall device associated hospitalizations occurred significantly more often in the TV-ICD group (TV-ICD: *n* = 18, 36.7% vs. S-ICD: *n* = 1, 4.8%; *p* < 0.001). There was no difference between groups for documented ventricular arrhythmias, ventricular arrhythmia episodes per patient or for appropriate or inappropriate shocks during FU. Anti-tachycardia pacing (ATP) was delivered to 9 (18.3%) of patients in the TV-ICD group but is not available from the S-ICD. During FU, no patient required a change of system for a bradycardia pacing in the S-ICD group or for cardiac resynchronization in either group. Outcome results did not differ between primary and secondary prophylactic indications for both cohorts. Hemodialysis was required in 3 (6.1%) and 2 (9.5%) patients in the respective TV-ICD and S-ICD groups without a significant difference (*p* = 0.318). All variables monitored during FU are shown in Table 2.

**Table 2:** Results from the follow-up.

	TV-ICD (49)	S-ICD (21)	p
Duration follow-up, months	55.1 (48.2–64.4)	55.4 (46.8–75.0)	0.790
Patients experiencing device-associated infection, *n* (%)	8 (16.3)	0	<0.05
Time to first device infection, months	29.2 ± 10.6	–	
Patients experiencing a CIED-associated complication, *n* (%)	13 (26.5)	1 (4.8)	<0.05
Lead related complications	10 (20.4)	0	<0.05
Patients with device associated hospitalization, *n* (%)	18 (36.7)	1 (4.8)	<0.001
Device associated hospitalizations per patient	0.44 ± 0.7	0.05 ± 0.2	<0.05
Patients with documented ventricular arrhythmia, *n* (%)	10 (20.4)	2 (9.5)	0.275
Number of ventricular arrhythmia episodes per patient	2.2 ± 2.9	0.4 ± 0.5	0.462
Patients receiving ATP, *n* (%)	9 (18.3)	0	<0.05
ATP per patient	0.2 ± 0.6	0	0.065
Patients receiving a shock, *n* (%)	8 (16.3)	1 (4.8)	0.191
Patients receiving adequate shocks, *n* (%)	6 (12.2)	1 (4.8)	0.157
Patients receiving inadequate shocks, *n* (%)	3 (6.0) Amount: 1, 3, 5	0	0.318
Patients receiving more than one shock, *n* (%)	2 (4.0) 3 and 5 shocks	1 (4.8) 2 shocks	0.458
Patients requiring hemodialysis during FU, *n* (%)	3 (6.1)	2 (9.5)	0.318

TV-ICD, transvenous ICD; S-ICD, subcutaneous ICD; ATP, anti-tachycardia pacing.

## Discussion

The aim of the present study was to investigate whether there was a difference in TV-ICD and S-ICD associated infections, complications and hospitalizations in patients with stage 4 CKD who received a prophylactic ICD.

Patients with progressive CKD exhibit an increased risk for heart failure, ventricular arrhythmias and sudden cardiac death (SCD) (3). This group of patients has been excluded from most studies demonstrating a reduced mortality for ICD compared with drug therapy (1, 14). Patients with progressive CKD (GFR <30 ml/min/1.73 m^2^) were excluded from the S-ICD Investigational Device Exemption Trial (15). Unsurprisingly, there are no specific recommendations in the ICD therapy guidelines for patients with progressive CKD.

The role of ICDs in this seriously compromised group of patients remains unclear as large studies failed to show a survival benefit from implanting ICDs in patients with CKD (16). Higher CIED infection rates are assumed to be a trigger for higher hospitalization rates in patients with CKD who received an ICD compared with control groups with CKD who did not receive an ICD (17). We therefore postulated that in a similar group of compromised patients, there would be a lower CIED infection and device related hospitalization rate in S-ICD recipients as there are no lead on or in the heart.

Baseline demographics are comparable to a cohort study of 5,877 patients with CKD and heart failure reported by Bansal et al. (16). However, the number of patients with coronary artery disease, history of myocardial infarction and ischemic cardiomyopathy is higher in our cohort whilst the age in our S-ICD group was significantly younger. Implanting physicians are more likely to choose an S-ICD in younger patients, as they expect fewer complications in patients with CKD due to preservation of the vasculature. In addition, there was a higher likelihood in younger patients for a further progression of the CKD with the need for hemodialysis (HD). The feasibility of creating an arteriovenous fistula in patients requiring HD can be complicated by the presence of a CIED in the vasculature. Venous occlusion or central venous stenosis has been reported in 11%–36% patients with transvenous CIED (18).

The age difference between our TV-ICD and S-ICD groups could be an explanation for a higher rate of atrial arrhythmia in the TV-ICD group and hence a higher proportion of patients receiving anti-arrhythmic medication and cardiac glycosides. With a higher incidence of atrial arrhythmias and anti-arrhythmic medication there can be concerns regarding a higher drug accumulation due to lower renal clearance (19–21). This could be an important issue regarding morbidity and mortality data in future investigations. The significant age difference in our study between both groups could favor the beneficial results for the S-ICD, however data from a TV-ICD meta-analysis and a S-ICD study suggest that device associated complications are not associated with higher age (22, 23).

Implantation duration of our groups was comparable with other large studies comparing TV-ICD and S-ICD therapies (24). Patients with progressive CKD experience a significantly higher proportion of intraoperative complications, especially bleeding, vascular injury and in-hospital mortality according to an analysis of a nationwide database from the United States of 40,075 cases with ICD implantation (25). Minor procedural complications occurred significantly more often in our TV-ICD cohort. The procedural complication rate of 4.8% in our S-ICD cohort was comparable to a 3.8% procedural complication rate of 429 S-ICD procedures reported by Knops et al. (24). The rate of procedural complications associated with TV-ICD implantation was higher in our study (12.2%) compared with a rate of 4.7% in 423 procedures reported by Knops et al. (24). A high rate of oral anti-coagulation in our TV-ICD cohort and a high rate of complications in patients with CKD receiving a TV-ICD are explanations (16). However, no difference between our groups for procedural complications requiring surgical or other interventions has been reported.

Patients with progressive CKD are at an exponentially increased risk for death with decreasing kidney function and increasing cardiovascular mortality being the leading cause of death, rather than renal failure (3, 26). Progressive CKD is associated with a wide range of cardiac and non-cardiac features providing a substrate for vulnerable myocardium and increased risk of arrhythmias and consequently SCD (27, 28). The incidence of ventricular arrhythmias together with adequate and inadequate therapy in both ICD groups, and their lack of significance, is comparable with a large cohort reported by Russo et al. (29). Device lifetime infection rate is 2%–3% for TV-ICD systems in a general population according to the Danish ICD registry (30). CKD is an independent risk factor for device infections (31). In a general population, CIED infections occur significantly more often in TV-ICD patients compared with S-ICD patients (29). If infections with S-ICD systems occur, these can frequently be handled conservatively with antibiotics without the need for removal, whereas TV-ICD systems need to be explanted as soon as possible, often with complicated TLE procedures (15, 32). Our data is in line with these reports as there were no infections in the intermuscularly implanted S-ICD group and a significantly higher proportion of 16.3% CIED infections in the TV-ICD group. The high prevalence of device associated infections in the TV-ICD group can be explained by the progressive CKD. In S-ICD patients with progressive CKD, even those with HD, do not have an increased incidence of CIED infections (33, 34). If however a device removal of an S-ICD would be required, the procedure is safe and easy to perform (35).

Patients with a TV-ICD system and CKD are not only exposed to a high risk of CIED infection, but also other device associated complications. Jukema et al. reported an overall TV-ICD associated complication rate in 80 (31.3%) patients with end-stage CKD, of which 4 (5.0%) patients required ICD removal for bacteremia during a median FU of 6.8 ± SD years (36). A hospitalization rate for device-associated complications of 36.7% in our TV-ICD groups seems reasonable for a high risk population considering a pooled complication-rate, excluding inappropriate therapy, of 9.1% in a large TV-ICD meta-analysis by Ezzat et al. during an average FU of 17.9 months and an annual rate of 12.0% for complications requiring surgical intervention in prospective registry reported by Hawkins et al. of 3410 ICD recipients in a general population during a median FU of 34 months (22, 37). A meta-analysis of 2,387 patients by Fong et al. reported that compared with TV-ICD patients, S-ICD patients experienced significantly fewer (RR: 0.14, 95% CI: 0.07–0.29, *p* < 0.0001) lead related complications (8). These findings are supported by our data showing significantly fewer CIED-associated complications, especially lead related complications, in the S-ICD group during a long-term FU.

### Limitations

Even though six centers participated, the sample size remains small. A population with a GFR between 15 and 29 ml/min/1.73 m^2^, an ICD indication as well as an expected good quality survival of more than one year is rare.

This study represents the only comparative analysis, albeit retrospective, of TV-ICD and S-ICD recipients with stage 4 CKD. The study was neither powered nor designed to investigate mortality or overall hospitalization rates. Due to small sample size we were only able to perform basic statistical analysis without multivariate analysis and adjustments. A large prospective, appropriately powered, randomized trial is required to verify the results presented in this study and draw further conclusions. Overall mortality and hospitalization in each of the ICD groups should be examined in the proposed trial as well as a third group of patients with the same baseline characteristics who do not receive an ICD.

## Conclusion

During a long-term follow-up of patients with stage 4 chronic kidney disease at high risk for ICD related complications, the intermuscularly implanted S-ICD has significantly fewer device-associated infections and complications compared with TV-ICDs.

## Data Availability

The raw data supporting the conclusions of this article will be made available by the authors, without undue reservation.
